# Reviewer Thank You

**DOI:** 10.1097/EE9.0000000000000376

**Published:** 2025-03-11

## Thank you, reviewers!

Without the help of countless reviewers, it would be impossible to publish a journal. This we all know. Now that *Environmental Epidemiology* has been around for some years, it is time to recognize the 369 reviewers who contributed to the journal’s success by submitting one or more reviews in 2021–2024. They are listed below in alphabetical order. Again, thanks!

Bert Brunekreef, PhD

Editor-in-Chief

Kjersti Aagaard

Sara Adar

Juan Aguilera

Anna Alari

Wael Al-Delaimy

Camilla Alvarez

Adeladza Amegah

Heresh Amini

Antonis Analitis

Zorana Andersen

Susan Anenberg

Thomas Arcury

Maria Argos

Victoria Arrandale

Richard Atkinson

Maryia Bakhtsiyarava

Kalpana Balakrishnan

Bridget Barker

Robert Barouki

Dana Barr

Dennis Barten

Xavier Basagana

Thomas Bateson

Mariska Bauwelinck

Hasan Bayram

Richard Belcher

Tom Bellander

David Bellinger

Kiros Berhane

Claudine Berr

Parveen Bhatti

Annibale Biggeri

Lizan Bloemsma

Annelise Blomberg

Michael Borghese

Joseph Braun

Carrie Breton

Harry Brown

Katrin Burkart

Jacob Burns

Igor Burstyn

Kim Cajachagua-Torres

Lucia Calderon

Carlos Cardenas

Christopher Carlsten

Lidia Casas

Joan Casey

Joan Casey

Aysegul Cebi

Giulia Cesaroni

Wei-Jen Chen

Weihong Chen

Renjie Chen

Jie Chen

Jeong Weon Choi

Tanya Christidis

Anil Chuturgoon

Cassie Clark

Birgit Claus Henn

Nathan Cohen

Thomas Cole-Hunter

Elena Colicino

Josh Colston

Laura Corlin

Brent Coull

Christine Cowie

Jim Crooks

Dan Crouse

Robert Dales

Jeroen de Bont

Kees de Hoogh

Audrey de Nazelle

Myrna de Rooij

Evan de Schrijver

Nina de Toro

Frank de Vocht

Stephanie DeFlorio-Barker

Evan Deschrijver

Nicole Deziel

Qian Di

Douglas Dockery

Wietske Dohmen

David Dosa

Jeroen Douwes

George Downward

Kristie Ebi

Marloes Eeftens

Stephanie Eick

Kathrin Engel

Shohreh Farzan

Daniela Fecht

Tomasso Filippini

Kelvin Fong

Francesco Forastiere

Meredith Franklin

Carmen Freire

Elaine Fuertes

Samuel Fuhrimann

Hannah Gardener

Antonio Gasparrini

Calvin Ge

Ulrike Gehring

Akhgar Ghassabian

Santu Ghosh

Mark Goldberg

Carlos Gould

Nelson Gouveia

Stephanie Grady

Lisa Grant Ludwig

James Grellier

Ariane Guilbert

Yuming Guo

Ivan Gutierrez-Avila

Monica Guxens

Omar Hahad

Simon Hales

Amber Hall

Frederik Hammes

Steve Hankey

Anna Hansell

Jaime Hart

Joachim Heinrich

Sarah Henderson

Michael Hendryx

Seulkee Heo

David Hernandez

Irva Hertz-Picciotto

Gerard Hoek

Barbara Hoffmann

Karen Holcomb

Christian Hoover

Jane Hoppin

Danny Houthuijs

Howard Hu

Wei Huang

Anke Huels

Ulla Hvidtfeldt

Carly Hyland

Sindana Ilango

Talat Islam

Benedicte Jacquemin

Bin Jalaludin

Nicole Janssen

Michael Jerrett

Hubertus Jersmann

Fay Johnston

Rena Jones

Kevin Josey

Andrea Joyce

Haidong Kan

Ravi Kant

Rebecca Kehm

Jules Kerckhoffs

Kenza Khomsi

Molly Kile

Patrick Kinney

Marianthi Kioumourtzoglou

Pirkka Kirjavainen

Milan Klawer

Jochem Klompmaker

Luke Knibbs

Bethany Knox

Manolis Kogevinas

Susan Korrick

Sara Kress

Norun Krog

Richard Kwok

Christine Ladd-Acosta

Francine Laden

Katrina Lambert

Eric Lavigne

Kaitlyn Lawrence

Ken Lee

Lauri Lehtimaki

Virissa Lenters

Naomi Letellier

Sherlock Li

Shanshan Li

Bessie Li

Zeyan Liew

Youn-Hee Lim

Bert Little

Petter Ljungman

Steffen Loft

Urszula Lopuszanska

Thomas Luben

Leire Luque Garcia

Heike Luttmann-Gibson

Roald Maes

Ebba Malmqvist

Alessandro Marcon

Sylvia Maritano

Pierre Masselot

Rob McConnell

Jasmine McDonald

Danielle Medgyesi

Erik Melen

Mark Mendell

Qi Meng

Mohamed Miri

Erika Mitchell

Katherine Moon

Jee-Young Moon

Brianna Moore

Geoff Morgan

Ali Moro

Hanns Moshammer

Matias Mrejen

Adetoun Mustapha

Rosemary Nabaweesi

Gabriele Nagel

Rajen Naidoo

Mrudula Naidu

Nathalia Naspolini

Tim Nawrot

David Nelson

Patricia Newcomb

Anne Nigra

Marie O’Neill

Pablo Orellano

Anton Orlov

Anna Oudin

Daniel Oudin

Kyriaki Papantoniou

Stefania Papatheodorou

Cynthia Parayiwa

Sung Kyun Park

Melissa Partyka

Marisa Patti

Neil Pearce

Meredith Pedde

Marie Pedersen

Jennifer Peel

Adjani Peralta

Zana Percy

Goran Pershagen

Cristian Perz-Fernandez

Susan Peters

Arthit Phosri

Marco Piccininni

Regina Pickford

Ajay Pillarisetti

Andrei Pyko

Xavier Querol

Arbor Quist

Ata Rafiee

Mostafijur Rahman

Divyangana Rakesh

Otavio Ranzani

Austin Rau

Khaiwal Ravindra

Matteo Renzi

Weeberb Requia

Ana Isabel Ribero

Hanadi Rifai

Beate Ritz

Harry Roels

Till Roenneberg

Eleanor Rogan

Megan Romano

Kathleen Ross

Isabell Rumrich

Mette Sorensen

Marc Saez

Evangelia Samoli

David Savitz

Lawrence Schell

Tamara Schikowski

Greet Schoeters

Jeremy Schraw

Jorg Schullehner

Brian Schwartz

Francesco Sera

Jessica Sexton

Lianne Sheppard

Mariana Simoes

Bhuchitra Singh

Robin Sinsamala

Claire Slesinski

Audrey Smargiassi

Lidwien Smit

Anna Smith

Michael Smolensky

Rina So

Johan Sommar

Ashley Song

Massimo Stafoggia

Jeffrey Stanaway

Heather Stapleton

Anne Starling

Katrin Steul

Jeanette Stingone

Kurt Straif

Maciek Strak

Jordi Sunyer

Katherine Svensson

Evelyn Talbott

Youran Tan

Anais Teyton

Jesse Thacher

Jeannette Therming Jorgensen

Darrin Thompson

George Thurston

Linwei Tian

Sandra Tilmon

Clara Amalie Gade Timmermann

Erik Timmermans

Aurelio Tobias

Shilu Tong

Jessica Trowbridge

Cecilie Uldbjerg

Annemoon van Erp

Ruth van Nispen

Marti van Tongeren

Berna van Wendel de Joode

Ana Maria Vicedo Cabrera

Veronica Vieira

Cristina Villanueva

Paul Villeneuve

Paolo Vineis

Jelle Vlaanderen

Julie von Behren

Katherine von Stackelberg

Judith Vonk

Trang VoPham

Donna Vorhees

Arthur Vranken

Danielle Wallace

Meng Wang

Cavin Ward-Caviness

Marcella Warner

Bertil Wegman

Scott Weichenthal

Gudrun Weinmayr

Marc Weisskopf

Gregory Wellenius

Amanda Wheeler

Samantha Williams

Augusta Williams

Kathrin Wolf

Christopher Worsham

Inge Wouters

Caradee Wright

Wentao Wu

Jun Wu

Shanshan Xu

Lisa Yamasaki

Xiaobo Yang

Jeff Yanosky

Lu Yao

Gyeyoon Yim

Peng Yin

Takashi Yorifuji

Astrid Zamora

Antonella Zanobetti

Hossein Zare

Ariana Zeka

Yu Zhang

Yan Zhang

Siqi Zhang

Shiyu Zhang

Boya Zhang

Charlie Zhong

Terry Zhou

Corwin Zigler

Jelle Zorn

Moniek Zuurbier

